# An evidence-based framework for priority clinical research questions for COVID-19

**DOI:** 10.7189/jogh.10-011001

**Published:** 2020-06

**Authors:** Carlyn Harris, Gail Carson, J Kenneth Baillie, Peter Horby, Harish Nair

**Affiliations:** 1 Emory University School of Medicine, Atlanta, Georgia, USA; 2 Centre for Global Health, Usher Institute, University of Edinburgh, Edinburgh, UK; 3 International Severe Acute Respiratory and Emerging Infection Consortium (ISARIC), Centre for Tropical Medicine and Global Health, University of Oxford, Oxford, UK; 4 Centre for Tropical Medicine and Global Health, University of Oxford, Oxford, UK; 5 Centre for Inflammation Research and Roslin Institute, University of Edinburgh, Edinburgh, UK

## Abstract

**Background:**

On 31 December, 2019, the World Health Organization China Country Office was informed of cases of pneumonia of unknown aetiology. Since then, there have been over 75 000 cases globally of the 2019 novel coronavirus (COVID-19), 2000 deaths, and over 14 000 cases recovered. Outbreaks of novel agents represent opportunities for clinical research to inform real-time public health action. In 2018, we conducted a systematic review to identify priority research questions for Severe Acute Respiratory Syndrome-related coronavirus (SARS-CoV) and Middle East Respiratory Syndrome-related coronavirus (MERS-CoV). Here, we review information available on COVID-19 and provide an evidenced-based framework for priority clinical research in the current outbreak.

**Methods:**

Three bibliographic databases were searched to identify clinical studies published on SARS-CoV and MERS-CoV in the outbreak setting. Studies were grouped thematically according to clinical research questions addressed. In February 2020, available information on COVID19 was reviewed and compared to the results of the SARS-CoV and MERS-CoV systematic review.

**Results:**

From the research objectives for SARS-CoV and MERS-CoV, ten themes in the literature were identified: Clinical characterisation, prognosis, diagnosis, clinical management, viral pathogenesis, epidemiological characterisation, infection prevention and control/transmission, susceptibility, psychosocial, and aetiology. For COVID19, some information on clinical presentation, diagnostic testing, and aetiology is available but many clinical research gaps have yet to be filled.

**Conclusions:**

Based on a systematic review of other severe coronaviruses, we summarise the state of clinical research for COVID-19, highlight the research gaps, and provide recommendations for the implementation of standardised protocols. Data based on internationally standardised protocols will inform clinical practice real-time.

On 31 December 2019, the World Health Organization China Country Office was informed of cases of pneumonia of unknown etiology detected in Wuhan City, China. Shortly after, the National Health Commission China reported that the outbreak was associated with exposures in a seafood market in Wuhan. Chinese authorities then isolated and identified a novel coronavirus and shared the genetic sequence on 12 January 2020 [1]. Phylogenetic analysis showed that the novel virus falls into the betacoronavirus family, with about 86% similarity to a bat SARS-like coronavirus [2]. Human-to-human transmission of the virus has been confirmed [3]. As of 18 February 2020, there have been 75 161 cases in 29 countries with 2008 deaths. Currently, the disease caused by 2019 novel coronavirus has been referred to as COVID-19, and the virus SARS-CoV-2.

Outbreaks, especially of novel agents, create a pressing need to collect data on clinical characterization, treatment, and validation of new diagnostics to inform rapid public health response. In 2018, we conducted a systematic review to identify the most common clinical research questions asked during outbreaks of SARS-CoV and MERS-CoV. We identified ten major clinical questions and provided recommendations for standardised protocol study designs that should be designed in the case of a new outbreak of a novel respiratory pathogen. Here, we review the currently available information on COVID-19 to determine which clinical questions from the systematic review findings have already been addressed, what information is lacking, and compare COVID-19 to SARS-CoV and MERS-CoV.

## METHODS

### Overview of methodology for 2018 SARS-CoV and MERS-CoV systematic review

We included any original study included conducted in a clinical setting during an acute outbreak of MERS and SARS but limited the SARS studies to those published during the SARS epidemic and (16 November 2002 through 5 July 2003) and 18 months thereafter to identify clinical questions relevant during the acute phases of the outbreak. Therefore, for SARS studies, the last publication date considered for full text review was 31 December 2004. There was no publication date restriction for MERS studies as outbreaks were ongoing.

Search terms to capture observational study designs such as cohort studies, cross-sectional studies, case-control studies, and case series were adapted from the Scottish Intercollegiate Guidelines Network (SIGN) search filters [4]. Search terms on diagnosis and prognosis were adapted from PubMed clinical query search filters provided in the PubMed Help manual [5]. To capture studies that were conducted in the epidemic or outbreak setting, subject headings such as “disease outbreaks”, “epidemics”, “pandemics”, etc. were included.

We applied our inclusion and exclusion criteria (**Table 1**) for both title and abstract screening and subsequently full text screening. Following data extraction, the objectives of the included studies were grouped thematically. Within each theme, articles with objectives that represented similar research questions were summarised.

**Table 1:** Inclusion and exclusion criteria for SARS-CoV and MERS-CoV systematic review

Inclusion criteria	Exclusion criteria
Studies must be:	1) the main objective was not the study of SARS-CoV or MERS-CoV
- Focused SARS-CoV or MERS-CoV	2) the study was not conducted primarily in a clinical setting (ie, population epidemiology studies, in-vitro studies, surveillance studies were excluded)
- Conducted in a clinical setting	3) the study was not conducted in an outbreak setting or did not analyse data that was collected in an outbreak setting
- Conducted on humans	4) non-human studies
Study designs considered: Observational studies (prospective cohort, retrospective cohort, case-control, case-series) and cross-sectional studies.	5) the sample size was less than 4
	6) the study was not original

### Methodology for review of the 2019 novel coronavirus

As this outbreak is in its initial stages, we reviewed resources such as: The World Health Organization Disease Outbreak News, Ministry of Health websites from affected countries, the Center for Disease Control’s Morbidity and Mortality Weekly report (MMWR), ProMED, and publications found on PubMed. For PubMed searches, the search term “novel AND coronavirus” was used, with a date range starting at 1 January 2020. The search was conducted on 20 February 2020. References of articles found through these searches were also reviewed. Only studies conducted in the clinical settings were included and single case reports were excluded. We compared our findings to the 2018 systematic review on SARS and MERS to determine which questions have already been addressed, what information is lacking, and provide recommendations for data sharing and clinical study designs to be conducted during the current outbreak. We grouped the results thematically from reviewing available resources on the novel coronavirus based on their relevance to the previously identified ten clinical questions.

### Role of the funding source

No funding body had any role in study design, in the collection, analysis, and interpretation of data; in the writing of the report; nor in the decision to submit the paper for publication.

## RESULTS

### 2018 SARS-CoV and MERS-CoV systematic review

A total of 124 studies (71% on SARS) were included for the final review and data extraction. All were conducted in a hospital setting. After thematically coding the objectives of the 124 studies, ten key themes emerged: clinical characterisation, prognosis, diagnosis, clinical management, viral pathogenesis, epidemiological characterisation, infection prevention and control/transmission, susceptibility, psychosocial, and aetiology. Originally, infection prevention and control and transmission were grouped separately. However, they were combined as most of the papers in the “transmission” category were concerned with transmission to hospital workers. Of note, only 16% of SARS articles were published before the end of the outbreak. **Table 2** defines the key clinical research questions identified from each theme. This table was modelled after a publication describing harmonisation of Zika virus protocols [6]. Themes appear in order based on how many SARS and MERS articles attempted to address a question within that theme.

**Table 2:** Themes and key clinical questions asked during outbreaks of SARS and MERS

Theme	Key question(s)	Objective
Clinical characterisation	What is the clinical presentation and spectrum of disease?	Determine symptomology, progression of disease, laboratory findings, and radiological features in different patient groups (adults vs children, immunosuppressed patients) and identify early presenting symptoms
Prognosis	What are the risk factors for death or severe illness?	Identify factors such as comorbidities, demographic factors, test parameters, etc. that predict death, ICU admission, etc.
Clinical management	What treatments are effective for MERS/SARS patients?	Determine the role of antiviral treatments, steroid treatments, or combination in comparison to supportive therapy
	What is the role of antivirals in treatment?	
	What is the role of steroids in treatment?	
Diagnosis	What is the optimal diagnostic test for detecting the virus?	Evaluate sensitivity/specificity/positive predictive value/negative predictive value of different diagnostic assays such as real-time RT-PCR and ELISA
Viral pathogenesis	What is the duration of viral shedding?	Determine the viral shedding profiles over time and in different body fluids
Epidemiological characterisation	What characteristics define a “case”?	Develop criteria for suspected, probable, and confirmed cases
Infection prevention and control / Transmission	What are the risk factors that pre-dispose health care workers to infection or transmission?	Determine the activities or prevention measures that are correlated with protection or infection in health care workers
Susceptibility	What are the risk factors for infection? (patient population)	Determine the risk factors for patient infection, in the community and health care setting
Psychosocial	What are the psychosocial consequences of infection with the virus?	Determine effect of illness, treatment, and isolation procedures on the psychological and social well-being of those infected

### COVID-19

#### Current state of knowledge for 2019-COVID-19

Here, we answer what clinical information is currently known about the novel coronavirus, using the above questions as a framework.

#### What is the clinical presentation and spectrum of disease?

The first aggregated patient data comes from a publication by Chaolin Huang and colleagues, who described clinical features of 41 admitted hospital patients, 6 of whom died. They collected data using an adaptation of International Severe Acute Respiratory and Emerging Infection Consortium’s (ISARIC) /World Health Organisation (WHO) internationally standardised data collection forms, updated for use with the novel coronavirus (https://isaric.tghn.org/novel-coronavirus/). They report that the novel coronavirus presentation resembles that of SARS: a viral pneumonia with fever, cough, dyspnoea, and fatigue. They also found a high concentration of cytokines in critically ill patients, compared to less severe cases [7]. Chen, Wang, and Kui et al. reported data for 99, 138, and 137 patients respectively. All reported similar symptoms to the Huang article: fever, cough, shortness of breath, and fatigue. Lymphocytopenia reported among all authors [8-10]. Chen et al. and Wang et al. also reported elevated lactate dehydrogenase levels in many patients. Wang et al. report that median hospital stay was 10 days for those discharged alive. Unlike SARS and MERS, there seems to be less presentation of gastrointestinal symptoms.

One report of pregnant patients demonstrated similar symptomology to non-pregnant patients. They also established that there was no evidence of vertical transmission to the child [11]. In the only study of infants with COVID-19, Wei et al. reported fever and upper respiratory symptoms common among nine infants. All infants were infected via family clusters [12]. Finally, radiological studies attempted to characterise the course of disease in chest imaging. Pan et al. report peak lung involvement at 10 days on CT studies [13]. Of note is a case series by Xie et al. that reported ground glass opacity in five patients with initial negative RT-PCR findings but suspected COVID-19. They were later confirmed to be infected [14]. Overall, clinical characterisation studies commonly found bilateral involvement with ground glass opacity, though a range of presentations were reported [8-10,15].

#### What are the risk factors for death or severe illness?

While risk factors for death and severe illness cannot be firmly established without large groups of patient data and multi-variable adjusted methods, some studies report similar risk factors among cohorts. The Novel Coronavirus Pneumonia Emergency Response Epidemiology Team in China analyzed data for over 44 000 patients [16]. Being over age 80, male, and having comorbid conditions were each associated with increased risk of mortality. In smaller studies, similar results were found. Chen et al., Wang et al., and Kui et al. report more cases of severe illness among older patients and patients with co-morbidities [8-10]. Chen et al. also report that characteristics of patients who died corresponded well with the MuLBSTA, a warning model for predicting morality in viral pneumonia [17]. It includes indexes such as multi-lobular infiltration, lymphopenia, bacterial co-infection, smoking, hypertension, and age.

#### What treatments are effective (including the roles of antivirals and steroids)?

In the publication by Chaolin Huang and colleagues, antibiotics and oseltamivir, and oxygen support were administered to some patients. Corticosteroids were used if patients were diagnosed with severe community acquired pneumonia [7]. Chen et al. reported antiviral, antibiotic, and non-invasive mechanical ventilation use but did not provide comparisons or suggestions for guidelines [8]. Kui et al. determined that use of systemic corticosteroids in their cohort did not show benefits, but early respiratory support improved outcomes [10]. In addition to a randomised controlled trial of lopinavir/ritonavir in adults hospitalised with COVID-19 (trial registration number: ChiCTR2000029308) [18], over 80 clinical trials are expected to determine therapeutic options [19].

#### What is the optimal diagnostic test for detecting the virus?

Several diagnostic tests for the novel coronavirus have been developed. Of note are those by Corman and colleagues [20] and by Hong Kong University School of Medicine [21]. The World Health Organization has provided preliminary guidance on specimen collection and shipment as well as reporting [22]. All information on diagnostic testing can be found at the WHO’s technical guidance site.

#### What is the duration of viral shedding?

Zou et al. obtained upper respiratory specimens from 18 patients in Zhuhai, China. Highest viral loads were detected soon after symptom onset, with higher loads in the nose and throat. They suggest that the shedding pattern of SARS-CoV-2 resembles patients with influenza. Asymptomatic patients were also found to have nasopharyngeal viral loads similar to symptomatic patients [23]. Additionally, Y. Zhang et al. found live virus in stool samples [24]. Zhou and colleagues have reported positive virus results from both oral swabs and bronchoalveolar lavage samples, while bronchoalveolar lavage samples yielded higher viral copies [25]. W. Zhang et al. report the presence of viral load in oral swabs, anal swabs, and blood, suggesting respiratory, fecal-oral, and blood transmission[26]. To et al. report serial saliva samples among 12 patients, with declining viral RNA levels after hospitalization. However, one patient showed viral shedding in saliva up to 11 days [27].

#### What characteristics define a “case”?

The World Health Organization has provided a case definition in their interim surveillance guidance for the novel coronavirus [28]. The definition includes patients with a severe acute respiratory infection (SARI) with relevant travel history within 14 days of illness or a health care worker caring for those with SARI.

#### What are the risk factors which pre-dispose health care workers to infection or transmission?

Currently, there are no data on what clinical activities are associated with an increased risk to health care workers. As of 14 February 2020, over 1700 health care workers have been infected [29]. The World Health Organization has provided interim guidelines for infection prevention and control [30].

#### What are the risk factors for infection in the patient population?

Currently, it is unclear whether certain demographics of the population are more susceptible to infection with the novel coronavirus. Most initial cases had contact with Wuhan’s Huanan Seafood Market, the suspected source of the outbreak, or had contact with those who had visited the market [1].

#### What is the causative agent of disease?

Sequencing analysis from lower respiratory tract samples from bronchoalveolar lavage identified the novel coronavirus. Genomes were released on GISAID.org. Due to similarities with related viruses, a bat reservoir is suspected [25].

#### What are the psychosocial consequences of infection with the virus?

Currently, there are no data on the psychosocial impacts of infection, hospitalization, or quarantine among affected patients and health care workers.

## DISCUSSION

We summarise the available clinical information on COVID-19, using results from a systematic review on SARS and MERS as a framework. As highlighted by David Heymann in a recent Lancet commentary, rapid action by clinicians and scientists to share data has made it possible to identify a causative agent, design and implement a diagnostic test, as well as begin to understand patient presentation [31]. As outlined above, there is much work to be done on understanding the clinical picture of the novel coronavirus. The World Health Organization R&D Research Blueprint and the Global Research Collaboration for Infectious Disease Preparedness (GLOPID-R) have identified similar research priorities as those we identified from our systematic review. Overlapping priorities include understanding viral pathogenesis, clinical characterisation studies, infection prevention and control, and candidate therapeutics [32].

To facilitate answering these key clinical questions, we suggest the following study designs (**Table 3**). Notably, the World Health Organization released a cases and contacts investigation protocol called “The First Few X (FFX)”. It involves prospective case finding and follow-up to gain an early understanding of key clinical, epidemiological, and virologic characteristics of the first cases of COVID-19 in a given country. They have also released a protocol to assess risk factors for infection among health care workers.

**Table 3:** Recommended study designs for COVID-19

Key question(s)	Recommended study design and associated resources
What is the clinical presentation and spectrum of disease?	Patient cohorts or patient registry.
	Resource for hospitalised cases: Modified ISARIC/WHO Clinical Characterization protocol using new CRF form for COVID-19 (https://isaric.tghn.org/novel-coronavirus/)
	The First Few X (FFX) WHO Protocol https://www.who.int/publications-detail/the-first-few-x-(ffx)-cases-and-contact-investigation-protocol-for-2019-novel-coronavirus-(2019-ncov)-infection)
What are the risk factors for death or severe illness?	Case-control study or prospective cohort with outcome of death or another defined poor outcome
What treatments are effective?	Randomised controlled trials or adaptive trial designs
What is the role of antivirals in treatment?	WHO Master Protocol: https://www.who.int/blueprint/priority-diseases/key-action/multicenter-adaptive-RCT-of-investigational-therapeutics-for-COVID-19.pdf?ua=1
What is the role of steroids in treatment?	
What is the optimal diagnostic test for detecting the virus?	Diagnostic validation studies with clinical specimens
What is the duration of viral shedding?	Prospective cohort with systematic serial collection of specimens
	Resource for hospitalised cases: ISARIC/WHO Clinical Characterization protocol (https://isaric.net/ccp)
What characteristics define a “case”?	Clinical or population-based cohorts
	Resource for hospitalised cases: ISARIC/WHO Clinical Characterization protocol (https://isaric.net/ccp)
	The First Few X (FFX) WHO Protocol
What are the risk factors which pre-dispose health care workers to infection or transmission?	HCW cohort studies or case-control studies
	Resource: WHO HCW risk factor protocol (https://www.who.int/publications-detail/protocol-for-assessment-of-potential-risk-factors-for-2019-novel-coronavirus-(2019-ncov)-infection-among-health-care-workers-in-a-health-care-setting)
What are the risk factors for infection? (patient population)	Patient cohort
	Resource: The First Few X (FFX) WHO Protocol
What are the psychosocial consequences of infection with the virus?	Mixed methods study (survey + qualitative interviews)
What is the causative agent of disease?	Laboratory based study with clinical specimens, fulfilling Koch’s postulates

### What is the clinical presentation and spectrum of disease?

In terms of clinical presentation, early recognition of symptoms and disease progression also allows for rapid isolation, early clinical care and limits onward transmission. WHO and ISARIC have released an updated version of their case report form (CRF) specifically for COVID-19. This can be used to collect anonymised, standardised clinical data to start to inform our understanding of the presentation and natural history of COVID-19. This CRF is being rolled out as a tool for public health and may or may not require ethical approval according to local regulations. A more in-depth characterisation, with the collection of serial biological samples through a research protocol and informed consent can be obtained through the ISARIC/WHO Clinical Characterization Protocol. This a standardised, prospective, observational study for the rapid investigation of patients with severe acute infections. The protocol was designed to characterise host and pathogen features, triage and treatment of disease [33]. The WHO FFX protocol, described above, may be used for the earliest cases to identify key clinical characteristics in real-time. The current clinical characterisation articles are a start to our understanding of the clinical presentation and spectrum of disease but much larger cohorts are needed for greater precision around estimates and to undertake prognostic and risk factor analyses. Based on the SARS and MERS systematic review, further clinical research in this area should include if and how symptomology, laboratory findings, and imaging studies differ between demographic groups (ie. adults vs children, immunosuppressed patients).

### What are risk factors for death or severe illness?

Based on recent data, male gender, advancing age and co morbidities seem to be associated with death and severe illness [16]. Understanding prognostic factors for death or severe illness helps hospitals and public health authorities determine resource allocation [34-36]. During the SARS epidemic, risk factors for mortality were advanced age, co-morbidities, and initial high inflammatory laboratory markers [37,38]. Similarly, for MERS, advanced age and co-morbidities are predictors of severe illness and death [39].

Many studies identified from the MERS and SARS systematic review were retrospective cohort studies and poor outcomes were assessed 21-30 days from symptom onset. In designing protocols for mortality or severe illness risk factors for COVID-19, case-control studies or prospective cohort studies should be used with an end-point at 90 days, so as not to miss late deaths. Whatever the optimal end-point for assessing outcome, standardising this outcome measure across studies will allow researchers to contribute to core data sets.

### What treatments are effective (including the role of antivirals and steroids)?

While there is some preliminary descriptive data on clinical management, randomized controlled trials are needed to determine the best treatment options for COVID-19. For now, the World Health Organization has issued interim guidelines for clinical management, adapted from their guidance for MERS-CoV. It includes recommendations for early recognition, early supportive therapy (oxygen, fluids, empirical antimicrobials) and against routine use of systemic corticosteroids unless indicated for another reason. They also provide guidelines for cases of septic shock [30]. Clinical trials to test therapeutic efficacy are ongoing, such as a trial of lopinavir/ritonavir (https://www.pharmaceutical-technology.com/analysis/coronavirus-mers-cov-drugs/). More studies are expected soon [19].

Most of studies identified in the SARS and MERS review were descriptive treatment studies. These observational studies are practical in the fast-paced outbreak setting, as they are easier than randomised controlled trials (RCTs) to design and require less administrative effort. However, when it comes to treatment, RCTs provide the best primary evidence for medical practice [40]. Among these studies, there was great heterogenicity for testing efficacy of specific treatments. This reflects the lack of global research coordination in delivering medical countermeasures during the MERS outbreaks and the SARS epidemic. This should be kept in mind when designing treatment protocols for COVID-19 and is being addressed by WHO.

The RCT on Ebola therapeutics in the Democratic Republic of the Congo is evidence that conducting clinical therapeutics research is possible in the context of an outbreak [41].

The WHO has released a Master Protocol for a multi-centre, adaptive, randomized, double-blind placebo-controlled clinical trial to evaluate safety and efficacy of therapeutic agents for the treatment of hospitalized patients with COVID-19. Using a Master Protocol across international sites can speed the implementation of clinical trials and quickly inform treatment options (https://www.who.int/blueprint/priority-diseases/key-action/multicenter-adaptive-RCT-of-investigational-therapeutics-for-COVID-19.pdf?ua=1).

### What is the optimal diagnostic test?

The rapid development of a diagnostic test for COVID-19 was a critical development and the result of international collaboration. As the outbreak progresses, it is important to continue monitoring diagnostic validity, such as sensitivity, specificity, positive predictive value, and negative predictive value. During an outbreak or epidemic, it is important to also validate diagnostic tests in low prevalence areas, as predictive values may change [42].

### What is the duration of viral shedding?

Understanding the duration of viral shedding and the shedding profile from different anatomical sites are key for both diagnosis and instituting infection prevention and control measures [43]. Current data suggest that SARS-CoV-2 viral loads are high at the beginning of symptom onset, are found in upper respiratory specimens and stool specimens, and are detectable in asymptomatic patients at levels similar to symptomatic patients [23]. One SARS study revealed that viral detection peaked at 2 weeks after onset for respiratory specimens and 2-3 weeks for stool and rectal specimens. The shedding peak in urine occurred around weeks 3-4. Rarely did patients shed virus 6 weeks after onset, however it was documented in a few stool specimens [44].

To evaluate the shedding profile during the COVID-19 outbreak, the ISARIC/WHO Clinical Characterization protocol can be adapted to prospectively and systematically collect serial samples from patients with suspected infection [33,45]. As with studies on SARS and MERS, serial samples should be collected from multiple body sites, including urine, faecal, and nasopharyngeal samples.

### What characteristics define a “case”?

In an outbreak, if multiple sites adopt a standardised protocol such as the ISARIC/WHO Clinical Characterization protocol to describe cases, case definitions could be created rapidly to inform accurate reports on incidence and prevalence. Developing criteria for confirmed cases is usually based on laboratory diagnosis. The World Health Organization has provided an interim case definition, and it will likely evolve as more data are shared on patient presentation.

### What risk factors pre-dispose health care workers to infection or transmission?

Over 1700 health care workers in Wuhan have been diagnosed with COVID-19 and there is no research available as to how this happened. Both MERS and SARS viruses showed nosocomial transmission amplification in the health care setting [46]. During the SARS epidemic, 21% of the infected patients were health care workers, and in some countries, this rate was as high as 50% [47]. Risk of SARS infection was associated with inconsistent use of personal protective equipment, and less than 2 hours of infection control training [48]. During the 2015 South Korea MERS outbreak, 44% of the 186 cases were patients that had been exposed to nosocomial transmission in hospitals, and 83% of total transmission events were due to five super spreading events in hospitals [49]. The recently released WHO protocol for evaluating risks to health care workers should be implemented as soon as possible to prevent future health care worker infections.

### What are the risk factors for infection (in patient population)?

The suspected major risk factors for COVID-19 are visiting the Wuhan market or being in contact with someone who had visited the market. The above-mentioned WHO FFX protocol can be used globally to assess risk factors for infection.

### What are the psychosocial consequences of infection with the virus?

In the 2018 SARS and MERS systematic review, only three studies with a psychosocial focus were identified. However, integrating social science research into clinical and epidemiological research during an outbreak can help inform the need for psychologists, psychiatrists, and social workers. Especially in diseases with human-to-human transmission, the effect of stigma and quarantine on mental health cannot be underestimated. Psychosocial manifestations can be explored with mixed methods studies.

### What is the causative agent of disease?

Because of rapid data sharing and laboratory protocols by the Chinese health officials and the World Health Organization, the causative agent of the novel coronavirus was rapidly identified.

### Limitations

Our systematic review summarises the questions that *are* answered in the context of new outbreaks, but this methodology cannot tell us what questions *should* be answered. However, we propose this as a proxy for ensuring key clinical questions are addressed early in an outbreak. We have summarised key knowledge gaps for COVID19, but we do not intend to suggest that this is an exhaustive list. Many of the most important discoveries hinge on the creativity and innovation to identify new questions. In this new outbreak, we need new ideas to build on the foundations that we have comprehensively summarised in this article.

### Implications for practice

The suggested study designs above can be used to inform standardised research protocols and define data sets that should be collected by hospitals around the world, if they are affected by COVID-19. As in any outbreak setting, priorities of local clinicians and public health authorities should be considered, especially in countries that integrate traditional medicine with Western medicine. Researchers may consider adopting a tiered approach as ISARIC has with the Clinical Characterisation protocols. The tiered approach allows sites to determine how much data or samples they can collect given their (limited) resources. This allows health care centres in low and middle-income countries to be represented in the data. Global solidarity is needed in the clinical research community as we may face the next pandemic of the 21st century. We all benefit from the data collected and shared. WHO have launched a clinical data collection platform for COVID-19 via the International Health Regulations and hope that member states will share their data. This uses an ISARIC WHO co-created CRF for COVID 19.

## CONCLUSIONS

Thanks to lessons learned from SARS and MERS, the international public health and research community has been able to rapidly respond to the emergence of this novel coronavirus. Based on a 2018 systematic review of SARS and MERS common clinical research questions, we provide a summary of the state of current clinical knowledge for the COVID-19, demonstrate what clinical research gaps still need to be filled, and provide recommendations on study designs. Many of the identified gaps, such as viral pathogenesis, clinical characterisation, infection prevention, and candidate therapeutics overlap with gaps identified the WHO’s R&D Blueprint research roadmap. If health care facilities around the world collect standardised patient data and quickly share it, it is likely that these core clinical research questions can be answered in real-time to inform clinical practices for COVID-19.

## References

[1] World Health Organization. Novel Coronavirus (2019-nCoV) SITUATION REPORT-1. Available: 2020https://www.who.int/docs/default-source/coronaviruse/situation-reports/20200121-sitrep-1-2019-ncov.pdf. Accessed: 20 February 2020.

[2] Zhu N Zhang D Wang W Li X Yang B Song J A novel coronavirus from patients with pneumonia in China, 2019. N Engl J Med. 2020;382:727-33. 10.1056/NEJMoa2001017 31978945 PMC7092803

[3] Chan JF-W Yuan S Kok K-H To KK-W Chu H Yang J A familial cluster of pneumonia associated with the 2019 novel coronavirus indicating person-to-person transmission: a study of a family cluster. Lancet. 2020;395:514-23. 10.1016/S0140-6736(20)30154-9 31986261 PMC7159286

[4] Scottish Intercollegiate Guidelines Network. Search filterns. 2018. Available: http://www.sign.ac.uk/search-filters.html. Accessed: 6 August 2018.

[5] National Center for Biotechnology InformationPubMed Help. Available: 2018 https://www.ncbi.nlm.nih.gov/books/NBK3827/. Accessed: 20 February 2020.

[6] Van Kerkove MD Reveiz L Souza JP Jaenisch T Carson G Broutet N Harmonisation of Zika virus research protocols to address key public health concerns. Lancet Glob Health. 2016;4:e911-2. 10.1016/S2214-109X(16)30255-8 27815145

[7] Huang C Wang Y Li X Ren L Zhao J Hu Y Clinical features of patients infected with 2019 novel coronavirus in Wuhan, China. Lancet. 2020;395:497-506. 10.1016/S0140-6736(20)30183-5 31986264 PMC7159299

[8] Chen N Zhou M Dong X Qu J Gong F Han Y Epidemiological and clinical characteristics of 99 cases of 2019 novel coronavirus pneumonia in Wuhan, China: a descriptive study. Lancet. 2020;395:507-13. 10.1016/S0140-6736(20)30211-7 32007143 PMC7135076

[9] Wang D Hu B Hu C Zhu F Liu X Zhang J Clinical characteristics of 138 hospitalized patients with 2019 novel coronavirus–infected pneumonia in Wuhan, China. JAMA. 2020; Feb 7. 10.1001/jama.2020.1585 32031570 PMC7042881

[10] Kui L Fang Y-Y Deng Y Liu W Wang M-F Ma J-P Clinical characteristics of novel coronavirus cases in tertiary hospitals in Hubei Province. Chin Med J (Engl). 2020;Feb 7. 32044814 10.1097/CM9.0000000000000744 PMC7147277

[11] Chen H Guo J Wang C Luo F Yu X Zhang W Clinical characteristics and intrauterine vertical transmission potential of COVID-19 infection in nine pregnant women: a retrospective review of medical records. Lancet. 2020;395:809-15. 10.1016/S0140-6736(20)30360-3 32151335 PMC7159281

[12] Wei M Yuan J Liu Y Fu T Yu X Zhang Z-J Novel Coronavirus infection in hospitalized infants under 1 year of age in China. JAMA. 2020;Feb 14. 10.1001/jama.2020.2131 32058570 PMC7042807

[13] Pan Y Guan H Zhou S Wang Y Li Q Zhu T Initial CT findings and temporal changes in patients with the novel coronavirus pneumonia (2019-nCoV): a study of 63 patients in Wuhan, China. Eur Radiol. 2020;Feb 13. 10.1007/s00330-020-06731-x 32055945 PMC7087663

[14] Xie X Zhong Z Zhao W Zheng C Wang F Liu J Chest CT for typical 2019-nCoV pneumonia: Relationship to negative RT-PCR testing. Radiology. 2020;Feb 12:200343. 10.1148/radiol.2020200343 32049601 PMC7233363

[15] Chang D Lin M Wei L Xie L Zhu G Dela Cruz CS Epidemiologic and clinical characteristics of novel coronavirus infections involving 13 patients outside Wuhan, China. JAMA. 2020;Feb 7. 10.1001/jama.2020.1623 32031568 PMC7042871

[16] The Novel Coronavirus Pneumonia Emergency Response Epidemiology Team The Epidemiological characteristics of an outbreak of 2019 novel coronavirus diseases (COVID-19) — China, 2020. China CDC Wkly. 2020;2:113-22. PMC8392929 34594836

[17] Guo L Wei D Zhang X Wu Y Li Q Zhou M Clinical features predicting mortality risk in patients with viral pneumonia: The MuLBSTA Score. Front Microbiol. 2019;10:2752. 10.3389/fmicb.2019.02752 31849894 PMC6901688

[18] Wang C Horby PW Hayden FG Gao GF A novel coronavirus outbreak of global health concern. Lancet. 2020;395:470-3. 10.1016/S0140-6736(20)30185-9 31986257 PMC7135038

[19] Maxmen A. More than 80 clinical trials launch to test coronavirus treatments. Nat. News. 2020. Available: https://www.nature.com/articles/d41586-020-00444-3. Accessed: 20 February 2020. 10.1038/d41586-020-00444-3 32071447

[20] Corman V, Bleicker T, Brunink S, Drosten C, Landt O, Koopmans M, et al. Diagnostic detection of 2019-nCoV by real-time RT-PCR. Berlin, Germany, 2020. Available: https://www.who.int/docs/default-source/coronaviruse/protocol-v2-1.pdf?sfvrsn=a9ef618c_2. Accessed: 20 February 2020.

[21] Poon L, Chu D, Peiris M. Detection of 2019 novel coronavirus (2019-nCoV) in suspected human cases by RT-PCR. Hong Kong, 2020. Available: https://www.who.int/docs/default-source/coronaviruse/peiris-protocol-16-1-20.pdf?sfvrsn=af1aac73_4. Accessed: 20 February 2020.

[22] World Health Organization. Laboratory testing for 2019 novel coronavirus (2019-nCoV) in suspected human cases Interim guidance. 2020. Available: https://www.who.int/health-topics/coronavirus/laboratory-diagnostics-for-novel-coronavirus. Accessed

[23] Zou L Ruan F Huang M Liang L Huang H Hong Z SARS-CoV-2 Viral load in upper respiratory specimens of infected patients. N Engl J Med. 2020;Feb 12. 10.1056/NEJMc2001737 32074444 PMC7121626

[24] Zhang Y Chen C Zhu S Shu C Wang D Song J Notes from the field isolation of 2019-nCoV from a stool specimen of a laboratory- confirmed case of the coronavirus disease 2019 (COVID-19). China CDC Wkly. 2020;2:2019-20. PMC8392928 34594837

[25] Zhou P, Yang X-L, Wang X-G, Hu B, Zhang L, Zhang W et al. Discovery of a novel coronavirus associated with the recent pneumonia outbreak in humans and its potential bat origin. bioRxiv. 2020;2020.01.22.914952.

[26] Zhang W Du R-H Li B Zheng X-S Yang X-L Hu B Molecular and serological investigation of 2019-nCoV infected patients: implication of multiple shedding routes. Emerg Microbes Infect. 2020;9:386-9. 10.1080/22221751.2020.1729071 32065057 PMC7048229

[27] To KK-W Tsang OT-Y Chik-Yan Yip C Chan K-H Wu T-C Chan JMC Consistent detection of 2019 novel coronavirus in saliva. Clin Infect Dis. 2020;Feb 12:ciaa149. 10.1093/cid/ciaa149 32047895 PMC7108139

[28] World Health Organization. Surveillance case definitions for human infection with novel coronavirus (nCoV) Interim guidance v1. Available: https://apps.who.int/iris/bitstream/handle/10665/330376/WHO-2019-nCoV-Surveillance-v2020.1-eng.pdf. Accessed: 20 February 2020.

[29] World Health Organization. Coronavirus disease 2019 (COVID-19) Situation Report – 25. 2020. Geneva: WHO; 2020.

[30] World Health Organization. Clinical management of severe acute respiratory infection when novel coronavirus (nCoV) infection is suspected. 2020. Available: https://www.who.int/docs/default-source/coronaviruse/clinical-management-of-novel-cov.pdf?sfvrsn=bc7da517_2&download=true. Accessed: 20 February 2020.

[31] Heymann DL Data sharing and outbreaks: best practice exemplified. Lancet. 2020;395:469-70. 10.1016/S0140-6736(20)30184-7 31986258 PMC7139409

[32] GloPID-R, World Health Organization R&D Blueprint. COVID 19 Public Health Emergency of International Concern (PHEIC) Global research and innovation forum: towards a research roadmap. 2020. Available: https://www.who.int/blueprint/priority-diseases/key-action/Global_Research_Forum_FINAL_VERSION_for_web_14_feb_2020.pdf?ua=1. Accessed: 20 February 2020.

[33] Dunning JW Merson L Rohde GGU Gao Z Semple MG Tran D Open source clinical science for emerging infections. Lancet Infect Dis. 2014;14:8-9. 10.1016/S1473-3099(13)70327-X 24355025 PMC7158987

[34] Fowler RA Lapinsky SE Hallett D Detsky AS Sibbald WJ Slutsky AS Critically ill patients with severe acute respiratory syndrome. JAMA. 2003;290:367-73. 10.1001/jama.290.3.367 12865378

[35] Alraddadi BM Al-Salmi HS Jacobs-Slifka K Slayton RB Estivariz CF Geller AI Risk factors for Middle East Respiratory Syndrome coronavirus infection among healthcare personnel. Emerg Infect Dis. 2016;22:1915-20. 10.3201/eid2211.160920 27767011 PMC5088034

[36] Tsui PT Kwok ML Yuen H Lai ST Severe acute respiratory syndrome: clinical outcome and prognostic correlates. Emerg Infect Dis. 2003;9:1064-9. 10.3201/eid0909.030362 14519241 PMC3016795

[37] World Health Organization. Consensus document on the epidemiology of severe acute respiratory syndrome (SARS). Geneva: WHO; 2003.

[38] Wang JT Sheng WH Fang CT Chen YC Wang JL Yu CJ Clinical manifestations, laboratory findings, and treatment outcomes of SARS patients. Emerg Infect Dis. 2004;10:818. 10.3201/eid1005.030640 15200814 PMC3323212

[39] Park J-E Jung S Kim A Park J-E MERS transmission and risk factors: a systematic review. BMC Public Health. 2018;18:574. 10.1186/s12889-018-5484-8 29716568 PMC5930778

[40] De Brun C. Finding the evidence: a key step in the information process. 2013.

[41] Mulangu S Dodd LE Davey RT Tshiani Mbaya O Proschan M Mukadi D A randomized, controlled trial of Ebola virus disease therapeutics. N Engl J Med. 2019;381:2293-303. 10.1056/NEJMoa1910993 31774950 PMC10680050

[42] Parikh R Mathai A Parikh S Chandra Sekhar G Thomas R Understanding and using sensitivity, specificity and predictive values. Indian J Ophthalmol. 2008;56:45-50. 10.4103/0301-4738.37595 18158403 PMC2636062

[43] Cheng PKC Wong DA Tong LKL Ip S-M Lo ACT Lau C-S Viral shedding patterns of coronavirus in patients with probable severe acute respiratory syndrome. Lancet. 2004;363:1699-700. 10.1016/S0140-6736(04)16255-7 15158632 PMC7112423

[44] Chan PKS To W-K Ng K-C Lam RKY Ng T-K Chan RCW Laboratory diagnosis of SARS. Emerg Infect Dis. 2004;10:825-31. 10.3201/eid1005.030682 15200815 PMC3323215

[45] ISARIC. Clinical Characterization Protocol. 2014. Available: https://isaric.tghn.org/protocols/clinical-characterization-protocol/. Accessed: 10 August 2018.

[46] Chowell G Abdirizak F Lee S Lee J Jung E Nishiura H Transmission characteristics of MERS and SARS in the healthcare setting: A comparative study. BMC Med. 2015;13:210. 10.1186/s12916-015-0450-0 26336062 PMC4558759

[47] Memish ZA. Chapter 55: SARS-Associated Coronavirus. In: Mehtar S (ed). Guide to Infection Control in the Hospital. International Society for Infectious Diseases. Brookline, Massachusettes; 2018.

[48] Lau JTF Fung KS Wong TW Kim JH Wong E Chung S SARS Transmission among hospital workers in Hong Kong. Emerg Infect Dis. 2004;10:280-6. 10.3201/eid1002.030534 15030698 PMC3322933

[49] Oh MD Park WB Park SW Choe PG Bang JH Song KH Middle east respiratory syndrome: What we learned from the 2015 outbreak in the republic of Korea. Korean J Intern Med. 2018;33:233-46. 10.3904/kjim.2018.031 29506344 PMC5840604

